# Parliament and the Profession

**Published:** 1918-05-18

**Authors:** 


### PARLIAMENT AND THE PROFESSION.

### Vaccination of Soldiers.

Mr. Macpherson stated that refusal by a soldier to be vaccinated is not an offence punishable under the Army Act. No officer is authorised to inflict punishment for such refusal. Soldiers voluntarily enlisted who, at the time of their enlistment, agreed in their attestation papers to be vaccinated, cannot be punished under the Army Act for refusing to be vaccinated.

### Botulism.

In reply to a question addressed to the President of the Local Government Board by Mr. Gilbert, Mr. Walsh said that there have been a few deaths from an obscure disease presenting nerve symptoms which resemble those of the very rare food disease, hitherto unknown in this country, called "botulism." The disease under investigation by the Local Government Board in collaboration with the Medical Research Committee. Up to the present inquiries have failed to associate the illness with the consumption of any particular article of food, and no special orders regarding food are at present contemplated.

### Indian Medical Service.

In reply to a question put by General Croft it was stated that accelerated promotion in the Indian Army was granted in consideration of the general acceleration of promotion in the combatant branches of the British Army. No similar ground exists for a general acceleration in the Indian Medical Service. As a special measure the Secretary of State for India in December last authorised the antedating of the promotion of certain captains of the Indian Medical Service with a view of securing for them a relative equality in seniority with that of corresponding officers of the Royal Army Medical Corps whose promotion had been specially accelerated by war conditions.

### Medical Students in the Navy.

Mr. Pretyman informed Mr. Watt that in January last an Order was issued to the Fleet calling for reports of the names of all officers and men (other than surgeon probationers) who have, as medical students, passed the first professional examination in chemistry, physics, and biology. On receipt of these reports, the lists are scrutinised, and, where such a course appears desirable, the officer or man is allowed to return to continue his studies. Surgeon probationers are allowed to be demobilised at the end of six months' service at home or twelve months' abroad in order to complete their studies and qualify.

### 1914 Star and Hospital Ships.

Mr. Pretyman informed Commander Craig that the award of the 1914 Star is not extended to cover casual landings such as may be made by the staffs of hospital ships in connection with their duties in the collection and embarkation of wounded. The same rule is followed by the War Office in respect of the Royal Army Medical Corps *personnel* of hospital ships.

